# Outcomes of early oseltamivir treatment for hospitalized adult patients with community-acquired influenza pneumonia

**DOI:** 10.1371/journal.pone.0261411

**Published:** 2021-12-15

**Authors:** Narongdet Kositpantawong, Smonrapat Surasombatpattana, Pisud Siripaitoon, Siripen Kanchanasuwan, Thanaporn Hortiwakul, Boonsri Charernmak, Ozioma Forstinus Nwabor, Sarunyou Chusri

**Affiliations:** 1 Infectious Disease Unit, Department of Internal Medicine, Faculty of Medicine, Prince of Songkla University, Hat Yai, Songkhla, Thailand; 2 Department of Pathology, Faculty of Medicine, Prince of Songkla University, Hat Yai, Songkhla, Thailand; Ohio State University Wexner Medical Center Department of Surgery, UNITED STATES

## Abstract

Early initiation of oseltamivir within 48 h to 5 days from illness onset has been associated with improved survival among patients with community-acquired influenza pneumonia. Delay of hospitalization limits early treatment and the survival of patients. To date, the effects of early oseltamivir initiation within 24 hours from admission on patient mortality has remained unknown. This retrospective study reviewed and analyzed the clinical and non-clinical outcomes of 143 patients, with community-acquired influenza pneumonia, who received oseltamivir within 24 h (group A) and after 24 h (group B) from admission. Among the patients, 82 (57.3%) received oseltamivir within 24 h while 61 (42.7%) received oseltamivir after 24 h. The median time from symptom onset to admission for group A and group B was not statistically significant (P < 0.001). The 14-day mortality rate was 9% and 23% for group A and B, respectively (P = 0.03), while the 30-day mortality were 15% and 30% for group A and B, respectively (P = 0.05). Administration of oseltamivir within 24 h significantly affected 30-day mortality rates (adjust OR: 0.14, 95% CI: 0.47–0.04, P < 0.01), particularly among patients with respiratory failure at admission (adjust OR: 0.08, 95% CI: 0+.30–0.06, P < 0.01). Survival analysis of patient with influenza pneumonia and respiratory failure at admission demonstrated significant difference between those who received oseltamivir within and after 24 h (*P* = 0.002). The results indicated that early oseltamivir initiation within 24 h improved the survival outcome mainly among those with respiratory failure at admission.

## Introduction

Influenza viral infection causes substantial morbidity and mortality as well as economic burden, particularly among patients with pneumonia [1]. The Center for Disease Control and Prevention estimated 9.4 million– 45 million cases of symptomatic illness due to influenza between 2010–2020, with annual hospitalization of 140,000–810,000 cases and 12,000–61,000 cases of mortality [2]. Mortality due to influenza pneumonia was highest among the elderly (>65 years), accounting for 62% of all influenza-related deaths. However, 32% of death occurred in adults aged 18–64 years [2]. This indicated that influenza-related deaths affect a wide age range of patients with influenza related pneumonia. Several studies have shown that influenza virus was the most prevalent among community-acquired viral pneumonia [3–5]. Treatment with neuraminidase inhibitors demonstrated clinical benefits including shortened duration of symptoms, low respiratory tract complication, reduced rate of hospitalization and shorter length of hospital stay [6–8]. Moreover, survival benefit was shown in hospitalized patients [9]. Identification of risk factors associated with severe pneumonia, and timely treatment improved the mortality outcome. The current recommendation is early testing and treatment of hospitalized patients who are suspected to have influenza related pneumonia with antiviral agents [10]. A meta-analysis reported that early treatment within 48h of symptom onset was associated with 55% and 38% reduction in mortality in adults and critically ill patients, compared with late treatment [11]. The overall mortality was not improved when treatments were initiated after 48h of symptoms onset. However, a 35% mortality risk reduction was noted for critically ill adult patients. Each day delay of antiviral initiation, up to a maximum of 5 days, increased the mortality hazard rate. In current practice, treatment is often delayed while awaiting results of laboratory tests [12]. A recent study found that the median time from onset of illness to hospitalization of patients with community-acquired influenza pneumonia was 5 days. Consequently, initiation of oseltamivir 5 days after symptom onset did not yield significant favorable outcomes among hospitalized patients [13]. The impact of initial oseltamivir treatment within 24 h has not been adequately investigated. Hence, the objective in this study is to determine the clinical outcome of early initiation of oseltamivir treatment within 24 h of hospitalization of adult patients with community-acquired influenza related pneumonia.

## Methods

### Study design and population

The study is a retrospective cohort study conducted among adult hospitalized patients in Songklanagarind hospital, an 800-bed university hospital located at the Prince of Songkla University in southern Thailand. Patients aged 18 years or above who were admitted with community-acquired influenza related pneumonia between January 2016 to December 2020 were included in this study.

Records of confirmed influenza cases were retrieved from the hospital information system and laboratory database. The revision of records was based on ICD10 as follows; J110 Influenza with pneumonia, virus not identified and J12 Viral pneumonia, not elsewhere classified. Patients with a diagnosis of influenza pneumonia via: 1. acute lower respiratory tract symptoms, 2. new pulmonary infiltration on chest radiographs and 3. positive samples of influenza virus infection tested by Sofia™ Influenza A + B FIA (Quidel Corporation, CA, USA) rapid influenza diagnostic test and/or VIASURE Flu A, B Real Time RT-PCR Detection Kit (CerTest Biotec S.L.). The viral RNA was extracted by magLEAD 12gC (Precision System Science Co., Matsudo, Japan). The PCR was performed on the CFX96 Touch Real-Time PCR Detection System. The M1 gene was detected at the cut-off threshold of 40 cycles in accordance with the manufacturer’s recommendation. Patients with initial bacterial coinfection were excluded from the final analysis. Early oseltamivir treatment was defined as receiving oseltamivir within 24 h from the time of admission. Oseltamivir dosage in this study was divided into high-dose (150 mg twice daily) and standard dose (75 mg twice daily).

### Data collection

Electronic medical records were retrospectively reviewed to obtain demographic data, comorbidities, symptoms onset, respiratory failure, APACHE II score, intensive care unit (ICU) admission, type of influenza, management including time of oseltamivir initiation, dosage, and duration of treatment, and clinical outcomes including 14-day, 30-day, and in-hospital mortality rates. Non-clinical outcomes included length of hospital stay and hospital costs. The patients with respiratory failure were defined as those who required mechanical ventilation during admission. We allocated the patients that received oseltamivir within 24 h in group A and patients that did not receive oseltamivir within 24 h into group B. The databases were accessed for analysis since December 2020 to April 2021.

### Statistical analysis

We compared the clinical characteristics and outcomes of group A and group B. Category variables are presented as number and percentages. Continuous variables are expressed as mean ± standard deviation in normal distribution and median ± interquatile rage (IQR) in non-normal distribution. Fisher’s exact test or Chi square was used for category variables. Student’s T test or Mann-Whitney U test was employed in regards to the continuous variable.

To compare between survivors and non-survivors, univariate analysis was used, factoring influencing clinical outcomes. Variables with P <0.2 from univariate or variables with clinical relevance were analyzed using a multivariate logistic regression model. A Cox proportional hazards model was used to analyze independent factors between survivors and non-survivors. A survival analysis between group A and group B, from the time of admission, was performed to assess the clinical outcome in both groups. The time started was defined as the day that influenza pneumonia was diagnosed on admission. The time ended was defined as the date that the patient outcome was documented, or the patient was excluded from the observation frame. All the analyses were performed using R software version 3.3.2 (R Foundation for Statistical Computing, Vienna, Austria).

### Ethical statement

Ethical approval was granted by the Ethics Committee of the Faculty of Medicine, Prince of Songkla University with the certification No. PSU EC: 55-141-14-1-3/ Sub 2. The data were fully anonymized before accessed and analyzed. According to the retrospective design of the study and relatively low risk for the participants, the need for informed consent was waived by the ethics committee.

## Results

**Fig 1:**
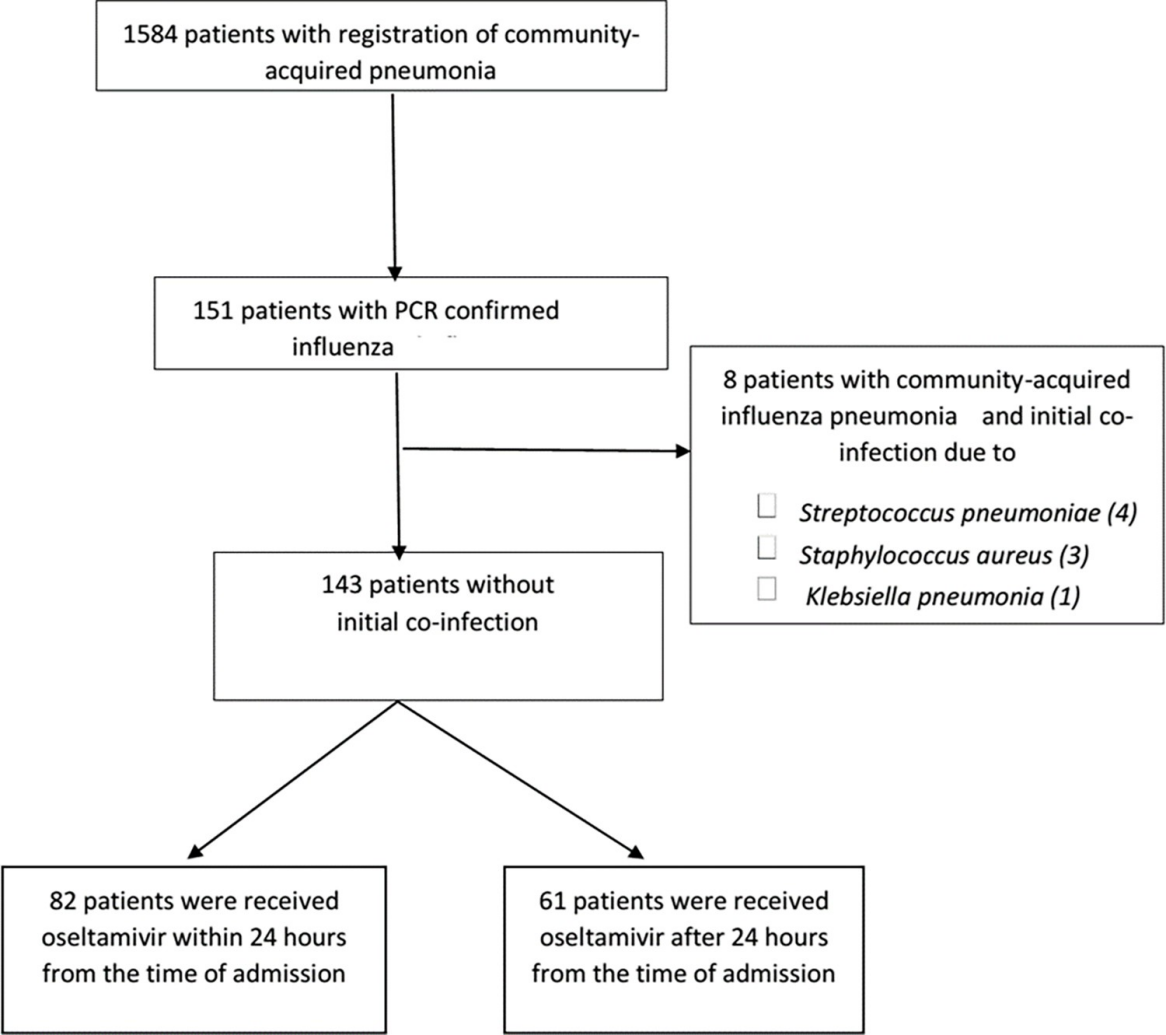
Flow chart of patient enrollment and inclusion in the study.

A total of 1584 patients were registered with community-acquired pneumonia in Songklanagarind Hospital, with 151 (9.5%) patients diagnosed of influenza pneumonia, and 127 (84%) patients had positive results of rapid test following positive result for PCR. There were 24 patients who only had positive PCR results. The study flow chart is presented in Fig 1. Eight patients who had bacterial pneumonia coinfection were excluded, while 143 patients were included in the analysis, consisting of 82 patients (57.3%) that received oseltamivir within 24 h from admission (group A) and 61 patients (46.7%) that received oseltamivir after 24 hours from admission (group B).

Demographic data, comorbidities, clinical characteristic, and treatment are summarized in the Table 1. There were no significant differences in patient characteristics between the two groups. The severity of patients in group A was significantly higher than group B. Higher proportion of the patients in group A had respiratory failure and initial ICU admission as well as higher APACHE II score. The median time from symptoms onset until admission in group A and group B was 6 days and 5 days, respectively but these were not statistically significant. The median time for oseltamivir initiation was 8 h for group A and 47 h for group B (P < 0.001).

**Table 1:** Comparison of clinical features between patients with community-acquired influenza pneumonia who received oseltamivir within 24 h from the time of admission and those that received later than 24 h.

Parameter	Patients who received oseltamivir within 24 h from the time of admission n = 82(%)	Patients who did not receive oseltamivir within 24 h from the time of admission n = 61(%)	*P-*value
**Demographics**			
Age (year), median (IQR)	45 (34,68)	45 (42,68)	0.509
Male sex	56 (68)	39 (64)	0.714
Current smoking	35 (43)	25 (41)	0.974
Comorbidities	64 (78)	42 (69)	0.294
Diabetes mellitus	29 (35)	21 (34)	0.999
Hypertension	33 (40)	31 (51)	0.277
Chronic kidney diseases	9 (11)	4 (7)	0.539
Cardiovascular diseases	14 (17)	7 (12)	0.486
Cerebrovascular diseases	2 (2)	1 (2)	0.999
Hematologic diseases	11 (13)	9 (15)	0.999
Malignancy	2 (2)	1 (2)	0.999
Chronic pulmonary airway diseases	25 (31)	20 (33)	0.912
Immunocompromised status	3 (4)	4 (7)	0.460
Obesity	46 (56)	34 (56)	0.999
**Clinical characteristic**			
Fever	68 (83)	51 (84)	0.999
Time from onset of symptoms to admission(day), median (IQR)	6(4,8)	5(4,7)	0.762
Respiratory failure at the admission	40 (49)	12 (20)	**< 0.001**
APACHEII score, median (IQR)	21 (19,24)	18 (16,22)	**0.036**
Initial admission in intensive care unit	49 (60)	10 (16)	**< 0.001**
Infection with type A influenza virus	42 (51)	33 (54)	0.864
**Treatment**			
Initiation of antibiotics within 24 hours	68 (83)	51 (84)	0.999
Time to initiate oseltamivir (hour), median (IQR)	8(4,17)	47(31,59)	**< 0.001**
Receiving high dosage of oseltamivir	62 (76)	49 (80)	0.641
Duration of oseltamivir(day), median (IQR)	10 (9,14)	10 (9,14)	0.932

IQR, interquartile range; APACHEII, Acute Physiology and Chronic Health Evaluation.

The comparison of outcomes between patients in group A and B is presented in Table 2. The 14-day and 30-day mortality rate were significantly lower in group A (*P* = 0.03 and *P* = 0.05, respectively). However, in-hospital mortality rate was indifferent between group A and group B. Bacterial superimposed infection was significantly lower in group A than in group B (*P* = 0.05). In addition, the non-clinical outcomes (length of hospital stay and hospital cost) were lower in group A than in group B (*P* = 0.05). In subgroup analysis among those who received high dosage of oseltamivir, 14-day, 30-day, in-hospital mortality rate and non-clinical outcomes were indifferent between the patients who received oseltamivir within 24 hours from time of admission and those who did not receive oseltamivir within 24 hours from time of admission. (S1 Table).

**Table 2:** Comparison of outcomes between patients with community-acquired influenza pneumonia who received oseltamivir within 24 h from the time of admission and those that received later than 24 h.

Outcome	Patients who received oseltamivir within 24 h from the time of admission n = 82(%)	Patients who did not receive oseltamivir within 24 h from the time of admission n = 61(%)	*P-*value
Clinical outcomes			
Mortality			
14-day	7 (9)	14 (23)	**0.030**
30-day	12 (15)	18 (30)	**0.050**
In-hospital	14 (17)	19 (31)	0.076
After the end of treatment with oseltamivir	7 (9)	7 (11)	0.560
Bacterial superimposed infection	12 (15)	18 (30)	**0.050**
Non-clinical outcomes			
Length of hospital stay after survival (days) [median (IQR)]	24 (16,34)	32 (20,35)	**0.021**
Cost (baht) [median (IQR)]			
Total hospital	156,333 (98,456–218,236)	184,882 (101,894–254,569)	**0.035**
Antimicrobial	23,889 (16,554–28,442)	31,222(27,887–34,002)	**< 0.001**
Non-antimicrobial	136,212 (100,654–199,221)	146,881 (121,488–211,956)	**0.039**

IQR, interquartile range.

In the subgroup analysis, the outcomes in patients with community-acquired influenza pneumonia who received oseltamivir within 48 h and after 48 h from the time of admission were not different (S2 Table). Interestingly, receiving oseltamivir within 24 hours from admission improved the mortality outcomes and non-clinical outcomes in patients presenting respiratory failure on admission (S3 Table). Among the patients without respiratory failure, early treatment within 24 hours from admission did not significantly decrease mortality outcome while it significantly shortened the length of hospital stay (S4 Table).

Table 3 presented the factors that influenced 30-day mortality among patients with community-acquired influenza pneumonia. Higher APACHE II score, initial respiratory failure at admission and timing of oseltamivir initiation (within 24 h) were significantly associated with 30-day mortality.

**Table 3:** Factors influencing 30-day mortality among 143 patients with community-acquired influenza pneumonia.

Variables	Values		Crude OR (95% CI)	Adjusted OR (95% CI)	*P-*value
	Survivors n = 113(%)	Non-survivors n = 30(%)			
Age (year) [median (IQR)]	45 (42,68)	45 (34,68)	1.19 (0.51,2.75)	1.07 (0.38,2.98)	0.899
Male sex	76 (67)	19 (63)	0.98 (2.38,0.36)	0.83 (2.94,0.24)	0.766
Underlying disease(s)	81 (72)	25 (83)	1.96 (5.55,0.69)	1.56 (5.55,0.43)	0.492
Current smoking	49 (43)	11 (37)	0.75 (1.72,0.33)	1.02 (2.70,0.38)	0.973
Immunocompromised status	6 (5)	1 (3)	0.61 (5.26,0.07)	0.26(3.44,0.02)	0.274
Obesity	59 (52)	21 (70)	2.13 (5.00,0.90)	2.04 (5.88,0.70)	0.178
APACHE II score [median (IQR)]	19 (15,21)	22 (19,22)	1.01 (0.97,1.54)	1.18 (1.03,1.33)	**0.048**
Initial intensive care unit admission	46 (41)	13 (43)	1.11 (2.44,0.49)	1.61 (4.76,0.53)	0.398
Respiratory failure at the admission	35 (31)	17 (57)	2.94 (6.66,1.28)	4.35(14.28,1.41)	**0.008**
Infection with type A influenza virus	55 (49)	20 (67)	2.11 (0.91,4.9)	2.12 (0.79,5.71)	0.081
Initiation of oseltamivir within 24 hours	70 (62)	12 (40)	0.41 (0.93,0.18)	0.14(0.47,0.04)	**< 0.001**
Initiation of antibiotics within 24 hours	92 (81)	27 (90)	2.04 (7.69,0.57)	2.04 (9.09,0.47)	0.325
Receiving high dosage of oseltamivir	89 (79)	22 (73)	0.74 (1.89,0.29)	1.01 (3.03,0.33)	0.996
Duration of antimicrobial agents (day) [median (IQR)]	10(9,14)	10(9,14)	1.01 (0.95,1.10)	1.00(0.91,1.04)	0.998

IQR, interquartile range; APACHEII, Acute Physiology and Chronic Health Evaluation; OR, Odds ratio; CI, Confidence interval.

**Fig 2:**
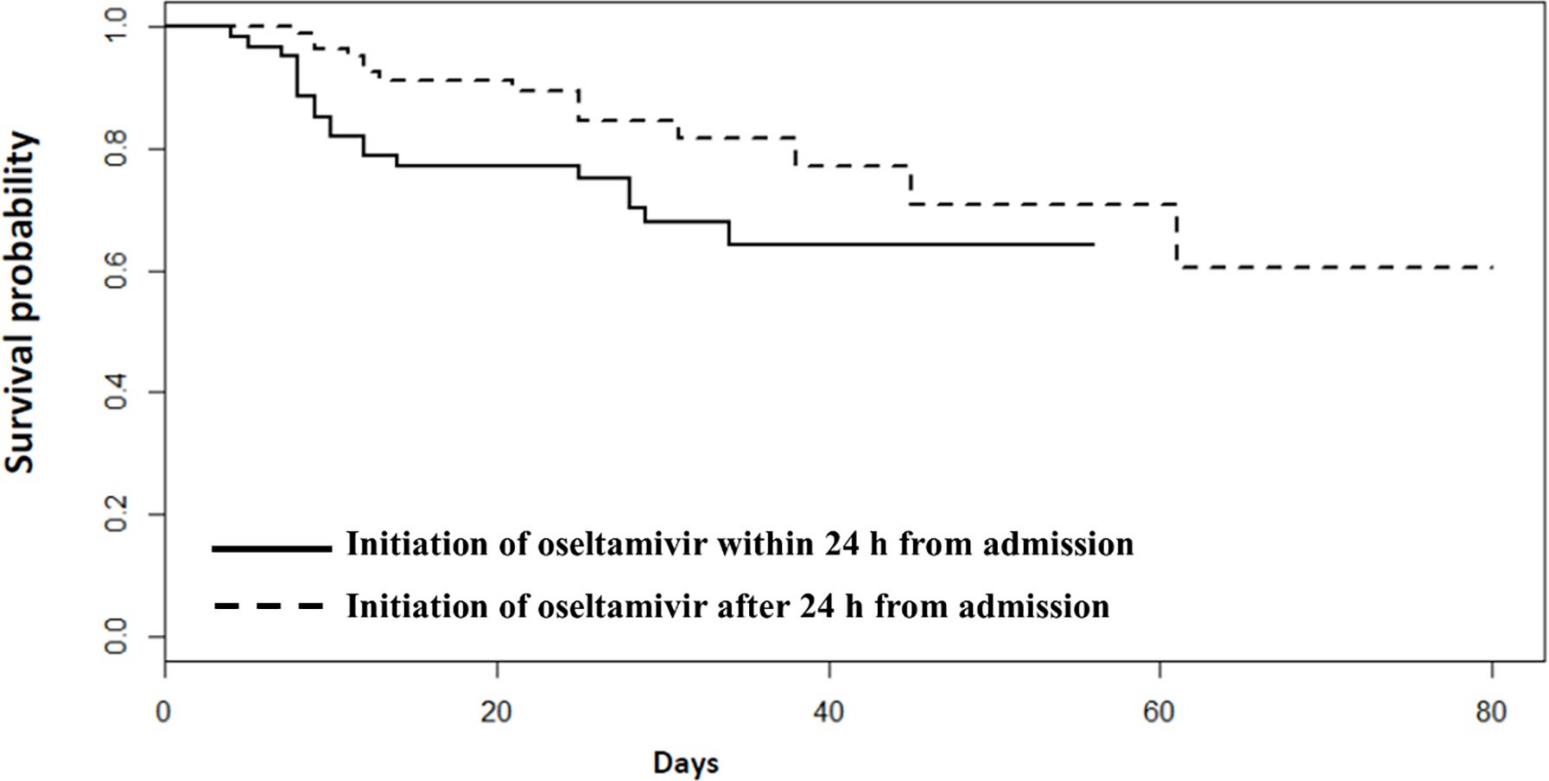
Survival between patients with community-acquired influenza pneumonia who received oseltamivir within 24 h from admission and those who received after 24 h from admission.

In the subgroup analysis, survival benefit was demonstrated in patients with respiratory failure who received oseltamivir within 24 h (S5 Table). Conversely, the early treatment effect was not revealed in patients without respiratory failure (S6 Table). Survival analysis of patients with community-acquired influenza pneumonia that received oseltamivir within 24 h from admission and those who received it later than 24 h demonstrated borderline different survival with *P* = 0.0579 by Log rank test (Fig 2). For patients with community-acquired influenza pneumonia and respiratory failure at admission, survival analysis for those who received oseltamivir within 24h of admission and those who received later than 24h demonstrated significant difference (*P* = 0.002) (S1 Fig), whereas for patients with community-acquired influenza pneumonia without respiratory failure at admission, survival analysis for those who received oseltamivir within 24 h of admission and those who received later than 24 h was not significantly different (*P* = 0.200) (S2 Fig).

## Discussion

In this study, we provided evidence of clinical and non-clinical benefits of the early initiation of oseltamivir within 24 h from admission among adult patients with community-acquired influenza pneumonia. Significantly lower 14-day and 30-day mortality were observed among patients who received oseltamivir within 24 h from admission. Similarly, significant difference was found among patients with respiratory failure at admission. However, this benefit was not observed among those without respiratory failure at admission. Delayed initiation of oseltamivir even within 48h after admission yielded no beneficial outcome. Our finding underscores that early treatment initiation within 24 h from admission has significant impact on outcome despite 5 days of symptom onset. Notably, mortality was significantly reduced notwithstanding the higher severity among patients who received early treatment.

Although neuraminidase inhibitors are effective against influenza, treatment outcomes are still debated [11, 14–17]. In a recent cohort study, early treatment with oseltamivir within 48 h was associated with lower mortality among critically ill patients that required ICU admission. However, beneficial outcomes were recorded for patients infected with influenza A/H3N2, but not for patients with influenza A/H1N2 and B [18]. The survival benefit of oseltamivir treatment within 5 days from illness onset has been demonstrated in several studies [9, 19–23]. A randomized control trial of empirical oseltamivir treatment within 24 h of hospitalization failed to reduce ICU transfer, re-hospitalization and 30-day mortality. Factors associated with clinical failure were possibly the small sample size of laboratory-confirmed influenza, associated lower respiratory tract infection, and low incidence of influenza [13].

In contrast, our results demonstrated the impact of early oseltamivir treatment within 24h of hospitalization despite the 6 days-interval between symptom onset and admission. Notably, oseltamivir treatment commenced within a median time of 8h in the early treatment group. To the best of our knowledge, this is the first study that demonstrates a survival benefit of early treatment within 24h from admission despite exceeding 5 days post illness onset. Moreover, most of the patients in this study presented with respiratory failure and required ICU attendance. The results are consistent with an observational study which reported a 33% reduction in ICU mortality following early oseltamivir treatment in critically ill patients [20], while the survival outcomes were insignificant in patients without respiratory failure. This finding agrees with a cohort study which reported that in-hospital mortality was not significantly reduced in patients with PaO2/FiO2 < 200 following oseltamivir treatment [21]. The incidence of influenza B was relatively high in the study population, but the results showed lesser oseltamivir efficacy on patients infected with influenza B as opposed to those infected with influenza A [24]. However, clinical benefits presented in this study were regardless of the type of influenza.

In this study, high doses of oseltamivir were used for critically ill cases. Although most studies have claimed that high-dose therapy was inadequate for the improvement of survival in a population mainly infected with influenza A [25–28]. Utilization of a high-dose regimen may be beneficial in critically ill cases with influenza B since the IC_50_ value was 10 times higher compared with influenza A [29]. Administration of high dose medication was reported to result in high plasma concentration. However, this benefit was not observed in our result. Our study demonstrated the effects of early treatment with oseltamivir in severely ill patients with influenza-related pneumonia. This study was based on analyzing observational data in regards to the treatment of hospitalized patients, differentiating the impact of early treatment between two groups, within 24 and within 48 h from admission. Mortality outcomes were not improved when treatment was initiated within 48 hours from admission.

There were several limitations in this study, that should be acknowledged. Firstly, similar to a previous observational study, a potential selection bias was introduced by different rates of influenza tests in each season [30]. Secondly, the circulating influenza in our studied population was equally proportionate between influenza A and B. Although in Thailand, influenza A is the prevalent type accounting for 76% in the tertiary care hospital system [31]. In a recent systematic review the epidemic strain was influenza A [32]. Our results may not claim an overall benefit in areas where influenza A is predominant. Thirdly, this study was conducted in a single tertiary care center, thus limiting its generalization to other level of healthcare services. Thus, in the setting of a tertiary care and referral center, the patients in this study had relatively severe manifestations with high APACHEII scores and relatively prolonged length of hospital stays. Fourthly, according to the nature of retrospective studies, the data on decision-making processes were insufficient thus an indication bias cannot be excluded. Fifthly, the number of patients in this study was relatively low. In this study, there was a 30-day mortality rate of 31% among the patients who did not receive oseltamivir within 24 h from the time of admission in this study and the enrolment of 143 patients only achieved a 54% power of prediction. Thus, the power of prediction among patients with initial respiratory failure and without initial respiratory failure was only at 55% and at 49%, respectively. Lastly, most of the patients in this study received high dosages of oseltamivir but in subgroup analysis among those who received high dosage of oseltamivir did not demonstrated the favourable outcomes among those who received oseltamivir within 24 hours from admission. Thus, in multivariate analysis, receiving high dosage of oseltamivir was not associated with survival benefit. Then, the results of this study did not inform the appropriate dosing of oseltamivir due to the observed variations.

## Conclusions

Treatment of patients with community-acquired influenza related pneumonia with oseltamivir within 24 h of hospital admission, despite exceeding 5 days from illness onset, led to a significant reduction in 14 and 30-day mortality and favorable non-clinical outcomes, particularly among patients with initial respiratory failure at admission.

## Supporting information

S1 Fig Survival between patients with community-acquired influenza pneumonia with respiratory failure at admission who received oseltamivir within 24 h from admission and those who received after 24 h from admission. (DOCX)

S2 Fig Survival between patients with community-acquired influenza pneumonia without respiratory failure at admission who received oseltamivir within 24 h from admission and those who received after 24 h from admission. (DOCX)

S1 Table Comparison of outcomes between patients with community-acquired influenza pneumonia at the admission who received high dosage of oseltamivir within 24 h and after 24 h from the time of admission. (DOCX)

S2 Table Comparison of outcomes between patients with community-acquired influenza pneumonia who received oseltamivir within 48 h and after 48 h from the time of admission. (DOCX)

S3 Table Comparison of outcomes between patients with community-acquired influenza pneumonia and respiratory failure at the admission who received oseltamivir within 24 h and after 24 h from the time of admission. (DOCX)

S4 Table Comparison of outcomes between patients with community-acquired influenza pneumonia without respiratory failure at admission who received oseltamivir within 24 h and after 24 h from the time of admission. (DOCX)

S5 Table Factors influencing 30-day mortality among 52 patients with community-acquired influenza pneumonia and respiratory failure at admission. (DOCX)

S6 Table Factors influencing 30-day mortality among 91 patients with community-acquired influenza pneumonia and without respiratory failure at admission. (DOCX)

## References

[1] CDC. Weekly US Influenza Surveillance Report. 2020.

[2] CDC. Disease burden of influenza: Centers of Disease Control and Prevention; 2020 [cited 2021 7th March]. Available from: https://www.cdc.gov/flu/about/burden/index.html.

[3] Radovanovic D, Sotgiu G, Jankovic M, Mahesh PA, Marcos PJ, Abdalla MI, et al. An international perspective on hospitalized patients with viral community-acquired pneumonia. European Journal of Internal Medicine. 2019;60:54–70. doi: 10.1016/j.ejim.2018.10.020 30401576 PMC7127340

[4] Alimi Y, Lim W, Lansbury L, Leonardi-Bee J, Nguyen-Van-Tam JS. Systematic review of respiratory viral pathogens identified in adults with community-acquired pneumonia in Europe. Journal of Clinical Virology. 2017;95:26–35. doi: 10.1016/j.jcv.2017.07.019 28837859 PMC7185624

[5] Burk M, El-Kersh K, Saad M, Wiemken T, Ramirez J, Cavallazzi R. Viral infection in community-acquired pneumonia: a systematic review and meta-analysis. European Respiratory Review. 2016;25(140):178–88. doi: 10.1183/16000617.0076-2015 27246595 PMC9487248

[6] Dobson J, Whitley RJ, Pocock S, Monto AS. Oseltamivir treatment for influenza in adults: a meta-analysis of randomised controlled trials. The Lancet. 2015;385(9979):1729–37. doi: 10.1016/S0140-6736(14)62449-1 25640810

[7] Treanor JJ, Hayden FG, Vrooman PS, Barbarash R, Bettis R, Riff D, et al. Efficacy and safety of the oral neuraminidase inhibitor oseltamivir in treating acute influenza: a randomized controlled trial. Jama. 2000;283(8):1016–24. doi: 10.1001/jama.283.8.1016 10697061

[8] Kaiser L, Wat C, Mills T, Mahoney P, Ward P, Hayden F. Impact of oseltamivir treatment on influenza-related lower respiratory tract complications and hospitalizations. Archives of Internal Medicine. 2003;163(14):1667–72. doi: 10.1001/archinte.163.14.1667 12885681

[9] Lee N, Choi K, Chan P, Hui D, Lui G, Wong B, et al. Outcomes of adults hospitalised with severe influenza. Thorax. 2010;65(6):510–5. doi: 10.1136/thx.2009.130799 20522848

[10] Uyeki TM, Bernstein HH, Bradley JS, Englund JA, File TM Jr, Fry AM, et al. Clinical practice guidelines by the Infectious Diseases Society of America: 2018 update on diagnosis, treatment, chemoprophylaxis, and institutional outbreak management of seasonal influenza. Clinical Infectious Diseases. 2019;68(6):e1–e47. doi: 10.1093/cid/ciy866 30566567 PMC6653685

[11] Muthuri SG, Venkatesan S, Myles PR, Leonardi-Bee J, Al Khuwaitir TS, Al Mamun A, et al. Effectiveness of neuraminidase inhibitors in reducing mortality in patients admitted to hospital with influenza A H1N1pdm09 virus infection: a meta-analysis of individual participant data. The Lancet Respiratory Medicine. 2014;2(5):395–404. doi: 10.1016/S2213-2600(14)70041-4 24815805 PMC6637757

[12] Appiah GD, Chaves SS, Kirley PD, Miller L, Meek J, Anderson E, et al. Increased antiviral treatment among hospitalized children and adults with laboratory-confirmed influenza, 2010–2015. Clinical Infectious Diseases. 2017;64(3):364–7. doi: 10.1093/cid/ciw745 28013261 PMC5480237

[13] Ramirez J, Peyrani P, Wiemken T, Chaves SS, Fry AM. A randomized study evaluating the effectiveness of oseltamivir initiated at the time of hospital admission in adults hospitalized with influenza-associated lower respiratory tract infections. Clinical Infectious Diseases. 2018;67(5):736–42. doi: 10.1093/cid/ciy163 29659754

[14] Neuraminidase inhibitors for influenza: a call for better research. Lancet. 2015;386(10003):1510 10.1016/S0140-6736(15)00523-1 26530602

[15] Gupta YK, Meenu M, Mohan P. The Tamiflu fiasco and lessons learnt. Indian Journal of Pharmacology. 2015;47(1):11. doi: 10.4103/0253-7613.150308 25821304 PMC4375804

[16] Jones M, Del Mar C, Hama R. Statistical and methodological concerns about the beneficial effect of neuraminidase inhibitors on mortality. The Lancet Respiratory Medicine. 2014;2(7):e9–e10. doi: 10.1016/S2213-2600(14)70126-2 24948431

[17] Hsu J, Santesso N, Mustafa R, Brozek J, Chen YL, Hopkins JP, et al. Antivirals for treatment of influenza: a systematic review and meta-analysis of observational studies. Annals of Internal Medicine. 2012;156(7):512–24. doi: 10.7326/0003-4819-156-7-201204030-00411 22371849 PMC6679687

[18] Lytras T, Mouratidou E, Andreopoulou A, Bonovas S, Tsiodras S. Effect of early oseltamivir treatment on mortality in critically ill patients with different types of influenza: a multiseason cohort study. Clinical Infectious Diseases. 2019;69(11):1896–902. doi: 10.1093/cid/ciz101 30753349

[19] Chen L, Han X, Li Y, Zhang C, Xing X. Impact of early neuraminidase inhibitor treatment on clinical outcomes in patients with influenza B-related pneumonia: a multicenter cohort study. Eur J Clin Microbiol Infect Dis. 2020:1–8. doi: 10.1007/s10096-020-03835-6 32026193

[20] Moreno G, Rodríguez A, Sole-Violán J, Martín-Loeches I, Díaz E, Bodí M, et al. Early oseltamivir treatment improves survival in critically ill patients with influenza pneumonia. ERJ Open Research. 2021. doi: 10.1183/23120541.00888-2020 33718494 PMC7938052

[21] Louie JK, Yang S, Acosta M, Yen C, Samuel MC, Schechter R, et al. Treatment with neuraminidase inhibitors for critically ill patients with influenza A (H1N1) pdm09. Clinical Infectious Diseases. 2012;55(9):1198–204. doi: 10.1093/cid/cis636 22843781 PMC12362346

[22] Groeneveld GH, Marbus SD, Ismail N, de Vries JJ, Schneeberger P, Oosterheert JJ, et al. Effectiveness of oseltamivir in reduction of complications and 30-day mortality in severe seasonal influenza infection. Int J Antimicrob Agents. 2020;56(5):106155. doi: 10.1016/j.ijantimicag.2020.106155 32898685

[23] Chaves SS, Pérez A, Miller L, Bennett NM, Bandyopadhyay A, Farley MM, et al. Impact of prompt influenza antiviral treatment on extended care needs after influenza hospitalization among community-dwelling older adults. Clinical Infectious Diseases. 2015;61(12):1807–14. doi: 10.1093/cid/civ733 26334053

[24] Baum SG. Oseltamivir and the influenza alphabet. The University of Chicago Press; 2006. 10.1086/505873 16838233

[25] Kiser T, Burnham E, Ho M, Moss M, Vandivier R. 656: Evaluation of high-dose versus standard-dose oseltamivir in critically ill patients with influenza. Critical Care Medicine. 2018;46(1):314.

[26] Noel ZR, Bastin MLT, Montgomery AA, Flannery AH. Comparison of high-dose versus standard dose oseltamivir in critically ill patients with influenza. Journal of Intensive Care Medicine. 2017;32(10):574–7. doi: 10.1177/0885066616638649 26992784

[27] Flannery AH, Thompson Bastin ML. Oseltamivir dosing in critically ill patients with severe influenza. Annals of Pharmacotherapy. 2014;48(8):1011–8. doi: 10.1177/1060028014535362 24816209

[28] Lee N, Hui D, Zuo Z, Ngai K, Lui G, Wo S, et al. A prospective intervention study on higher-dose oseltamivir treatment in adults hospitalized with influenza a and B infections. Clinical Infectious Diseases. 2013;57(11):1511–9. doi: 10.1093/cid/cit597 24046309

[29] McKimm-Breschkin J, Trivedi T, Hampson A, Hay A, Klimov A, Tashiro M, et al. Neuraminidase sequence analysis and susceptibilities of influenza virus clinical isolates to zanamivir and oseltamivir. Antimicrobial Agents and Chemotherapy. 2003;47(7):2264–72. doi: 10.1128/AAC.47.7.2264-2272.2003 12821478 PMC161875

[30] Lindegren ML, Griffin MR, Williams JV, Edwards KM, Zhu Y, Mitchel E, et al. Antiviral treatment among older adults hospitalized with influenza, 2006–2012. PLoS One. 2015;10(3):e0121952. doi: 10.1371/journal.pone.0121952 25807314 PMC4373943

[31] Narong N, Manajit S, Athipanyasil S, Athipanyasilp N, Sutthent R, Kantakamalakul W, et al. Prevalence of Influenza Virus Type and Subtype at Siriraj Hospital, Bangkok, Thailand During 2013–2017. Ramathibodi Medical Journal. 2020;43(3):1–7.

[32] Caini S, Kroneman M, Wiegers T, El Guerche‐Séblain C, Paget J. Clinical characteristics and severity of influenza infections by virus type, subtype, and lineage: a systematic literature review. Influenza and Other Respiratory Viruses. 2018;12(6):780–92. doi: 10.1111/irv.12575 29858537 PMC6185883

