# Supplementary figures and images for: Outcomes of early oseltamivir treatment for hospitalized adult patients with community-acquired influenza pneumonia
Source: PLoS One. 2021 Dec 15;16(12):e0261411. doi: 10.1371/journal.pone.0261411 (PMC8673668; doi:10.1371/journal.pone.0261411)

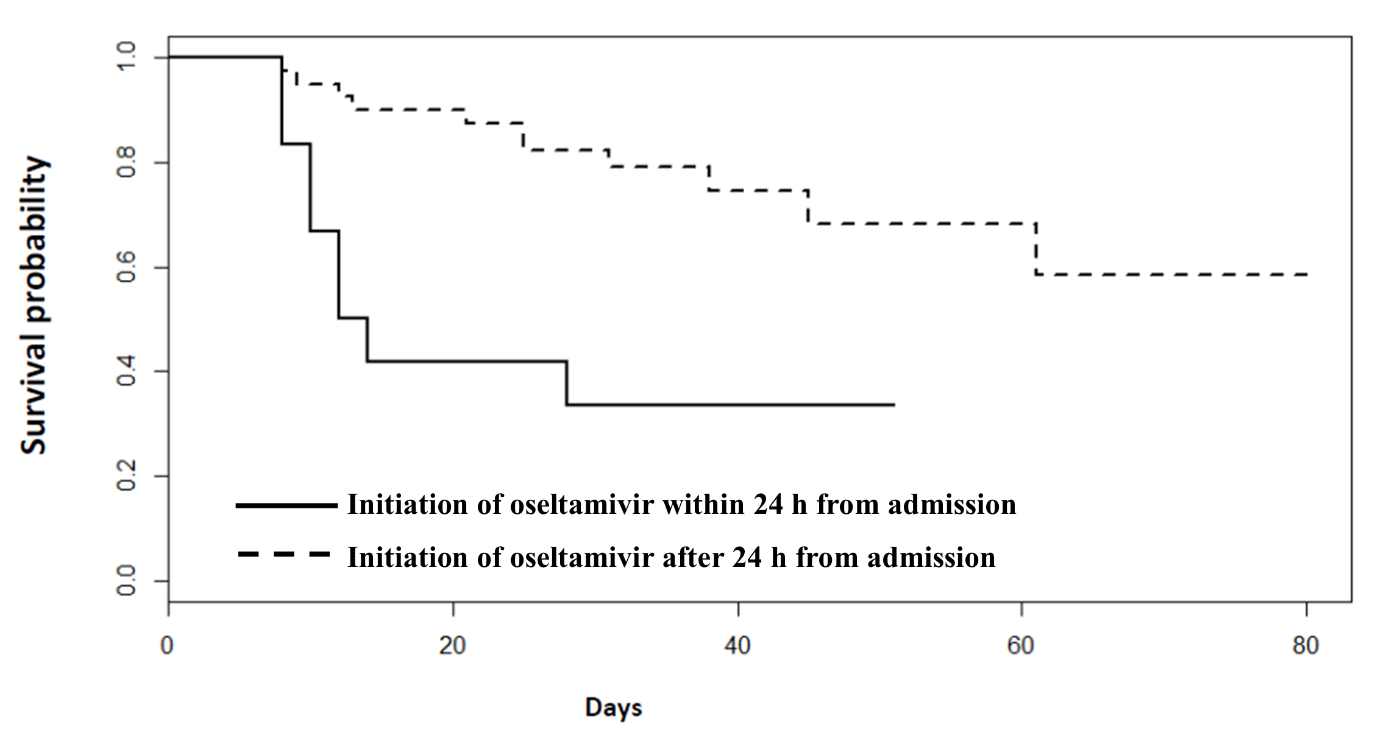


**S1 Fig.**

Supplement: S1 Fig — (DOCX) [file pone.0261411.s001.docx]

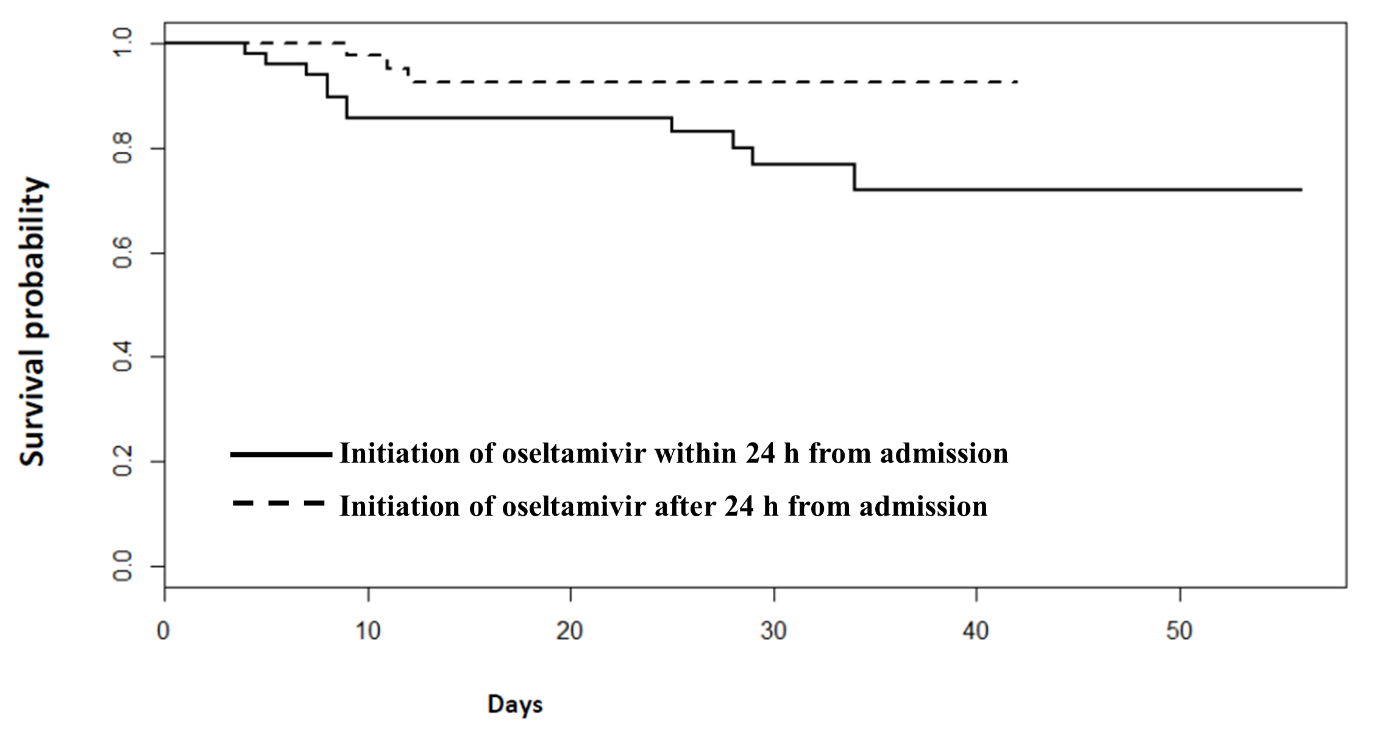


**S2 Fig.**

Supplement: S2 Fig — (DOCX) [file pone.0261411.s002.docx]
