# Supplementary material for: Outcomes of early oseltamivir treatment for hospitalized adult patients with community-acquired influenza pneumonia
Source: PLoS One. 2021 Dec 15;16(12):e0261411. doi: 10.1371/journal.pone.0261411 (PMC8673668; doi:10.1371/journal.pone.0261411)
Supplement: S1 Table — (DOCX) [file pone.0261411.s003.docx]

**S1 Table**

| Outcome | Patients who received high dosage of oseltamivir within 24 hours from the time of admission (n=62)(%) | Patients who did not received high dosage of oseltamivir within 24 hours from the time of admission (n=49) (%) | *P-*value |
| --- | --- | --- | --- |
| Clinical outcomes |  |  |  |
| Mortality |  |  |  |
| 14-day | 7 (11) | 12 (24) | 0.075 |
| 30-day | 9 (15) | 13 (26) | 0.121 |
| In-hospital | 13 (21) | 17 (35) | 0.110 |
| After the end of treatment with oseltamivir | 6(10) | 6(12) | 0.666 |
| Bacterial superimposed infection | 11 (18) | 16 (32) | 0.074 |
| Non-clinical outcomes |  |  |  |
| Length of hospital stay after survival (days) [median (IQR)] | 27 (20,35) | 29 (21,35) | 0.064 |
| Cost (baht) [median (IQR)] |  |  |  |
| Total hospital | 174,885 (100,221-211,556) | 179,456 (102,745-255,856) | 0.081 |
| Antimicrobial | 31,236(27,723-45,653) | 32,934(26,898-46,008) | 0.674 |
| Non-antimicrobial | 141,235 (75,654-178,365) | 149,885 (85,441-185,123) | 0.068 |
