# Supplementary material for: Outcomes of early oseltamivir treatment for hospitalized adult patients with community-acquired influenza pneumonia
Source: PLoS One. 2021 Dec 15;16(12):e0261411. doi: 10.1371/journal.pone.0261411 (PMC8673668; doi:10.1371/journal.pone.0261411)
Supplement: S2 Table — (DOCX) [file pone.0261411.s004.docx]

**S2 Table**

| Outcome | Patients who received oseltamivir within 48 hours from the time of admission (n=94)(%) | Patients who did not received oseltamivir within 48 hours from the time of admission (n=49) (%) | *P-*value |
| --- | --- | --- | --- |
| Clinical outcomes |  |  |  |
| Mortality |  |  |  |
| 14-day | 15 (16) | 6 (12) | 0.729 |
| 30-day | 22 (23) | 8 (16) | 0.441 |
| In-hospital | 24 (26) | 9 (18) | 0.450 |
| After the end of treatment with oseltamivir | 12(13) | 7(14) | 0.799 |
| Bacterial superimposed infection | 12 (13) | 8 (16) | 0.559 |
| Non-clinical outcomes |  |  |  |
| Length of hospital stay after survival (days) [median (IQR)] | 29 (23,37) | 30 (24,39) | 0.675 |
| Cost (baht) [median (IQR)] |  |  |  |
| Total hospital | 175,674 (107,453-222,065) | 180,452 (108,332-263,442) | 0.057 |
| Antimicrobial | 31,342(24,889-36,553) | 30,934(24,001-35,672) | 0.672 |
| Non-antimicrobial | 142,884 (98,117-197,023) | 150,212 (100,354-208,675) | 0.051 |

IQR, interquartile range.
