# Supplementary material for: Outcomes of early oseltamivir treatment for hospitalized adult patients with community-acquired influenza pneumonia
Source: PLoS One. 2021 Dec 15;16(12):e0261411. doi: 10.1371/journal.pone.0261411 (PMC8673668; doi:10.1371/journal.pone.0261411)
Supplement: S3 Table — (DOCX) [file pone.0261411.s005.docx]

**S3 Table**

| Outcome | Patients who received oseltamivir within 24 hours from the time of admission (n=40) (%) | Patients who did not receive oseltamivir within 24 hours from the time of admission (n=12) (%) | *P-*value |
| --- | --- | --- | --- |
| Clinical outcomes |  |  |  |
| Mortality |  |  |  |
| 14-day | 4 (10) | 7 (58) | **0.001** |
| 30-day | 9 (23) | 8 (67) | **0.011** |
| In-hospital | 11 (28) | 8 (67) | **0.019** |
| After the end of treatment with oseltamivir | 7 (18) | 2 (17) | 0.947 |
| Bacterial superimposed infection | 11 (28) | 7(58) | **0.033** |
| Non-clinical outcomes |  |  |  |
| Length of hospital stay after survival (days) [median (IQR)] | 31 (25,39) | 40(34,57) | **0.016** |
| Cost (baht) [median (IQR)] |  |  |  |
| Total hospital | 201,478 (137,666-256,882) | 284,798 (153,774-307,117) | **0.026** |
| Antimicrobial | 29,567 (19,885-32,965) | 36,991(28,116-36,364) | **0.004** |
| Non-antimicrobial | 162,987 (123,985-221,145) | 196,447 (142,878-287,365) | **0.031** |
