# Supplementary material for: Outcomes of early oseltamivir treatment for hospitalized adult patients with community-acquired influenza pneumonia
Source: PLoS One. 2021 Dec 15;16(12):e0261411. doi: 10.1371/journal.pone.0261411 (PMC8673668; doi:10.1371/journal.pone.0261411)
Supplement: S4 Table — (DOCX) [file pone.0261411.s006.docx]

**S4 Table**

| Outcome | Patients who received oseltamivir within 24 hours from the time of admission (n=42) (%) | Patients who did not receive oseltamivir within 24 hours from the time of admission (n=49) (%) | *P-*value |
| --- | --- | --- | --- |
| Clinical outcomes |  |  |  |
| Mortality |  |  |  |
| 14-day | 3 (7) | 7 (14) | 0.331 |
| 30-day | 3 (7) | 10(20) | 0.133 |
| In-hospital | 3 (7) | 11 (22) | 0.084 |
| After the end of treatment with oseltamivir | 1(2) | 4 (8) | 0.262 |
| Bacterial superimposed infection | 10(24) | 12(24) | 0.940 |
| Non-clinical outcomes |  |  |  |
| Length of hospital stay after survival (days) [median (IQR)] | 17(13,28) | 24 (19,29) | **0.042** |
| Cost (baht) [median (IQR)] |  |  |  |
| Total hospital | 87,236 (66,879-105,888) | 96,354 (70,889-112,365) | **0.044** |
| Antimicrobial | 16,987 (12,321-19,965) | 17,974(12,900-20,004) | 0.785 |
| Non-antimicrobial | 52,369 (48,567-79,365) | 68,227 (59,224-98,745) | **0.031** |
