# Supplementary material for: Outcomes of early oseltamivir treatment for hospitalized adult patients with community-acquired influenza pneumonia
Source: PLoS One. 2021 Dec 15;16(12):e0261411. doi: 10.1371/journal.pone.0261411 (PMC8673668; doi:10.1371/journal.pone.0261411)
Supplement: S5 Table — (DOCX) [file pone.0261411.s007.docx]

**S5 Table**

| Variables | Values | | Crude OR (95% CI) | Adjusted OR (95% CI) | *P-*value |
| --- | --- | --- | --- | --- | --- |
|  | Survivors (*n* = 35) (%) | Non-survivors (*n* = 17) (%) |  |  |  |
| Age (years) [median (IQR)] | 44 (42,62) | 67(42,75) | 1.02 (1.05,0.99) | 1.06 (1.12,1.01) | **0.009** |
| Male sex | 23 (66) | 10 (59) | 0.75 (2.43,0.23) | 2.70 (3.33,0.25) | 0.396 |
| Underlying disease(s) | 34 (97) | 15 (88) | 0.22 (2.63,0.02) | 2.04 (100,0.06) | 0.694 |
| Current smoking | 13 (37) | 4 (24) | 0.52 (1.92,0.14) | 0.52 (4.16,0.07) | 0.537 |
| Obesity | 28 (80) | 12 (71) | 0.59 (2.27,0.16) | 7.69 (100,0.39) | 0.148 |
| APACHE II score [median (IQR)] | 21 (18,24) | 24 (20,26) | 1.17 (0.98, 1.22) | 1.05 (1.01,1.12) | **0.046** |
| Initial intensive care unit admission | 19 (54) | 11 (65) | 1.54 (5.00,0.47) | 2.77 (16.67,0.48) | 0.233 |
| Infection with type A influenza virus | 15 (43) | 12 (71) | 3.2 (0.93,11.05) | 5.25 (0.68,40.6) | 0.091 |
| Initiation of oseltamivir within 24 hours | 31 (89) | 9 (53) | 0.14 (0.59,0.03) | 0.08 (0.30,0.064) | **< 0.001** |
| Initiation of antibiotics within 24 hours | 27 (77) | 16 (94) | 4.76 (50,0.54) | 5.88 (100,0.35) | 0.172 |
| Receiving high dosage of oseltamivir | 27 (77) | 13 (77) | 0.96 (3.84,0.24) | 5.26 (100,0.37) | 0.183 |
| Duration of antimicrobial agents(day)[median (IQR)] | 10(9,14) | 10(9,14) | 1.09 (0.84,1.19) | 1.02(0.81,1.09) | 0.975 |

IQR, interquartile range; APACHEII, Acute Physiology and Chronic Health Evaluation; OR, Odds ratio; CI, Confidence interval.
