# Supplementary material for: Outcomes of early oseltamivir treatment for hospitalized adult patients with community-acquired influenza pneumonia
Source: PLoS One. 2021 Dec 15;16(12):e0261411. doi: 10.1371/journal.pone.0261411 (PMC8673668; doi:10.1371/journal.pone.0261411)
Supplement: S6 Table — (DOCX) [file pone.0261411.s008.docx]

**S6 Table**

| Variables | Values | | Crude OR (95% CI) | Adjusted OR (95% CI) | *P-*value |
| --- | --- | --- | --- | --- | --- |
|  | Survivors (*n* = 78) (%) | Non-survivors (*n* = 13) (%) |  |  |  |
| Age (years) [median (IQR)] | 42 (25,45) | 45 (42,68) | 0.96 (1.12,0.92) | 0.93 (1.15,0.88) | 0.059 |
| Male sex | 53 (68) | 9 (69) | 1.06 (3.85,0.30) | 0.95 (4.54,0.20) | 0.952 |
| Underlying disease(s) | 47 (60) | 10 (77) | 2.02 (8.33,0.56) | 1.61 (8.33,0.31) | 0.563 |
| Current smoking | 36 (46) | 7 (54) | 1.37 (4.35,0.41) | 1.72 (7.69,0.38) | 0.426 |
| Obesity | 31 (40) | 9 (69) | 3.45 (12.5,0.96) | 5.00 (25.00,0.93) | 0.060 |
| APACHE II score [median (IQR)] | 16 (12,19) | 19 (16,21) | 1.01 (0.97,1.54) | 1.18 (1.03,1.33) | **0.050** |
| Initial intensive care unit admission | 27 (35) | 2 (15) | 0.34 (1.67,0.07) | 0.44 (4.00,0.05) | 0.450 |
| Infection with type A influenza virus | 40 (51) | 8 (62) | 1.52 (0.46,5.06) | 1.38 (0.32,5.92) | 0.661 |
| Initiation of oseltamivir within 24 hours | 39 (50) | 3 (23) | 0.30 (1.18,0.08) | 0.23 (1.45,0.04) | 0.096 |
| Initiation of antibiotics within 24 hours | 65 (83) | 11 (85) | 1.09 (5.55,0.22) | 1.26 (9.09,0.18) | 0.806 |
| Receiving high dosage of oseltamivir | 62 (80) | 9 (69) | 0.58 (2.13,0.16) | 1.00 (5.00,0.20) | 0.996 |
| Duration of antimicrobial agents(day)[median (IQR)] | 10(9,14) | 10(9,14) | 0.96 (1.03,0.88) | 0.99(1.11,0.93) | 0.987 |

IQR, interquartile range; APACHEII, Acute Physiology and Chronic Health Evaluation; OR, Odds ratio; CI, Confidence interval.
